# Improving the structure-function relationship in glaucomatous and normative eyes by incorporating photoreceptor layer thickness

**DOI:** 10.1038/s41598-018-28821-z

**Published:** 2018-07-11

**Authors:** Masato Matsuura, Yuri Fujino, Takashi Kanamoto, Hiroshi Murata, Mieko Yanagisawa, Kazunori Hirasawa, Tatsuya Inoue, Nobuyuki Shoji, Kenji Inoue, Junkichi Yamagami, Ryo Asaoka

**Affiliations:** 1 Department of Ophthalmology, The University of Tokyo, Tokyo, Japan; 2 Department of Ophthalmology, Graduate school of Medical Science, Kitasato University, Sagamihara, Kanagawa Japan; 3 Department of Ophthalmology, Hiroshima Memorial Hospital, Hiroshima, Japan; 4 NHR Biomedical Research Centre for Ophthalmology, Moorfields Eye Hospital NHS Foundation Trust and UCL Institute of Ophthalmology, London, United Kingdom; 5 Inouye Eye Hospital, Tokyo, Japan; 6 Department of Ophthalmology, JR Tokyo General Hospital, Tokyo, Japan

## Abstract

The purpose of the study was to investigate whether the structure-function relationship in glaucomatous and normative eyes is improved by considering photoreceptor layer thickness. Humphrey 10-2 visual fields (VF) and optical coherence tomography were carried out in 615 eyes of 391 subjects, including 100 eyes of 53 healthy controls and 515 eyes of 338 glaucoma patients. The relationship between mean VF sensitivity and the thickness of the retinal nerve fiber layer (RNFL) and ganglion cell layer and inner plexiform layer (GCL + IPL) was analyzed using linear mixed models, by glaucoma status and degree of myopia. The structure-function relationship was also analyzed by supplementing the RNFL and GCL + IPL thickness with the thicknesses of: (i) the inner nuclear layer and outer plexiform layer (INL + OPL); (ii) the outer nuclear layer and inner segment of photoreceptor layer (ONL + ISL); (iii) the outer segment layer of photoreceptor and retinal pigment epithelium (OSL + RPE). The model included total thickness of RNFL, GCL + IPL and OSL + RPE was highly more optimal than the model that only included the total thickness of RNFL and GCL + IPL, in all subsets of eyes by glaucoma status and degree of myopia.

## Introduction

Glaucoma is characterized by progressive degeneration of retinal ganglion cells (RGCs). These cells receive visual information from photoreceptors (PhRs) and collectively transmit the information to the brain through the optic disc and retinal nerve fiber layer (RNFL). Many studies have investigated the structure-function relationship in glaucoma using optical coherence tomography (OCT), assessing damage to RGCs and the RNFL^1–4^. It remains controversial whether PhR thickness is important for the structure-function relationship; recent studies investigating OCT-measured thickness of the PhR layer have failed to show that a decline in this measurement is associated with the advance of glaucoma^5–8^. These studies investigated the relationship between PhR thickness and visual field (VF) sensitivity using the 24-2 or 30-2 Humphrey Field Analyzer (HFA, Carl Zeiss Meditec, Dublin, CA) test pattern, however, the macular scan region of OCT corresponds to a much narrower area of the VF^9,10^. Thus, it may be more appropriate to measure the relationship between macular PhR layer thickness and VF sensitivity using a 10 degrees VF test pattern, rather than the 24-2 or 30-2 HFA test pattern.

In our previous report, we investigated the structure-function relationship between OCT measured retinal thicknesses and the central 10 degrees VF; we concluded that it was beneficial to consider the PhR’s outer segment layer (OSL) thickness in order to improve the structure-function relationship^11^. However, this result was investigated only in eyes with glaucoma and it is unclear whether a similar relationship can be observed in normative eyes. The structure-function relationship is important in normative eyes because it has been reported that there is a significant correlation between the thickness of ganglion cell layer (GCL)+ inner plexiform layer (IPL) and VF sensitivity^12^. Furthermore, retinal thickness is decreased in eyes with high myopia due to the elongation of the eye^13–16^; therefore, it is also important to investigate the effect of OSL thickness on the structure-function relationship in highly myopic eyes with and without glaucoma.

In this study we investigate the structure-function relationship in normative and glaucomatous eyes with and without high myopia. Structural measurements from OCT were compared with functional measurements from the HFA 10-2 VF test, and we investigate whether the structure-function relationship is improved by considering OSL thickness in addition to the thicknesses of RNFL and GCL.

## Methods

This study was approved by the Research Ethics Committee of Graduate School of Medicine and Faculty of Medicine at the University of Tokyo, Inouye Eye Hospital, JR Tokyo General Hospital and Hiroshima Memorial Hospital. Written consent was given by patients for their information to be stored in the hospital database and used for research; otherwise, based on the regulations of the Japanese Guidelines for Epidemiologic Study 2008 issued by the Japanese Government, the study protocols did not require that each patient provide written informed consent. Instead the protocol was posted at the outpatient clinic to notify participants of the study. This study was performed according to the tenets of the Declaration of Helsinki.

### Subjects

Subjects comprised 615 eyes of 391 subjects, including 100 normative eyes of 53 subjects (26 eyes of 14 subjects were highly myopic and the remaining 74 eyes of 39 subjects were not highly myopic), and 515 eyes with primary open angle glaucoma (POAG) of 338 patients (159 eyes of 107 subjects were highly myopic and remaining 356 eyes of 231 subjects were not highly myopic). All eyes underwent complete ophthalmic examinations, including biomicroscopy, gonioscopy, intraocular pressure measurement, funduscopy, refraction, best-corrected visual acuity measurement, axial length (AL) measurement, as well as OCT imaging and VF testing. All study participants were enrolled between the period of April 2013 and September 2017 at either the University of Tokyo Hospital, Inouye Eye hospital, JR Tokyo General Hospital or Hiroshima Memorial Hospital.

Primary open-angle glaucoma (POAG) was defined as (1) presence of typical glaucomatous changes in the optic nerve head such as a rim notch with a rim width ≤0.1 disc diameters or a vertical cup-to-disc ratio of >0.7 and/or a retinal nerve fiber layer defect with its edge at the optic nerve head margin greater than a major retinal vessel, diverging in an arcuate or wedge shape; (2) gonioscopically wide open angles of grade 3 or 4 based on the Shaffer classification; (3) aged between 20 and 80 years old; (4) eyes with visual acuity ≧0.5 LogMAR; and (5) refractive error <+3.0 diopter. Exclusion criteria were possible secondary ocular hypertension in either eye, pseudophakic eyes, people with other systemic or ocular disorders that could affect the study results.

Inclusion criteria for the normal group were; (1) no abnormal findings, except for clinically insignificant senile cataract, on biomicroscopy, gonioscopy, and funduscopy, (2) no history of ocular diseases that could affect the results of OCT examinations, such as diabetic retinopathy or age-related macular degeneration, (3) aged between 20 and 80 years old, (4) normal VF test results according to the Anderson-Patella criteria^17^, and (5) refractive error <+3.0 diopter. Eyes with anomalous discs were cautiously excluded. Pseudophakic eyes were also excluded.

### VF testing

VF testing was performed, within three months of the OCT examination, using the HFA with the SITA Standard strategy and the Goldmann III target. Normative and glaucomatous eyes were measured using the 10-2 program. Near refractive correction was used as necessary. All of the participants had previous experience in VF examinations and unreliable VFs defined as fixation losses greater than 33%, false-positive responses greater than 33%, or false-negative greater than 33% were excluded. In HFA, the mean deviation (MD) is calculated from the total deviation values, however, it is weighted toward the center of the VF, which is not appropriate in the current study because we wanted to investigate the relationship between retinal layer thickness on OCT and visual function. Thus, the unweighted mean of the VF threshold values (mTH) was calculated and used in subsequent analyses.

### SD-OCT measurement

**Figure 1:**
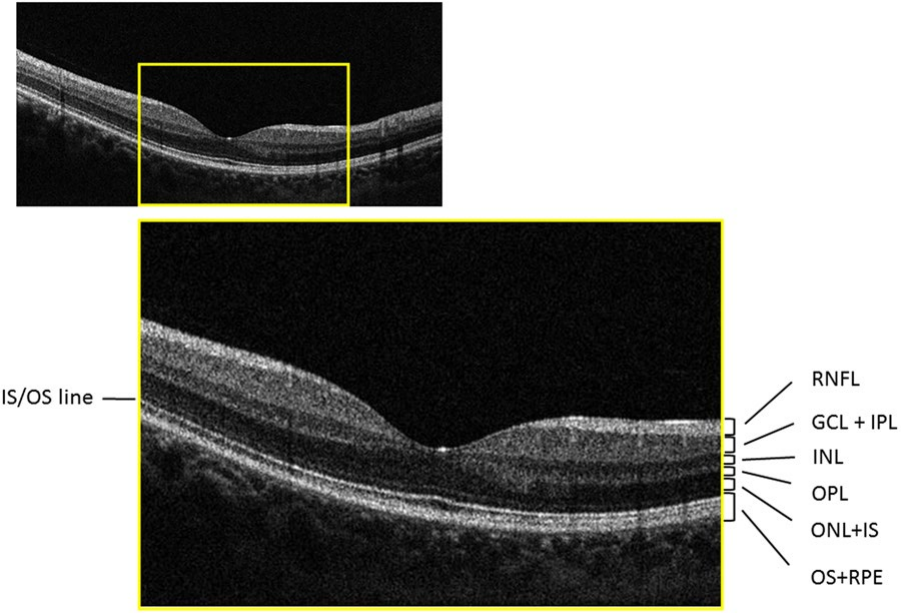
Macular retinal layer thickness measurements in a sample case. The layers of RNFL, GCL + IPL, INL, OPL, ONL + ISL, OSL + RPE are illustrated in a sample case (38 years old, female, normal). RNFL: retinal nerve fiber layer, GCL + IPL: ganglion cell layer and inner plexiform layer, INL: inner nuclear layer, OPL: outer plexiform layer, ONL + ISL: outer nuclear layer and inner photoreceptor layer segment, OSL + RPE: outer photoreceptor segment and retinal pigment epithelial.

SD-OCT data were obtained using the RS 3000 (Nidek Co ltd., Aichi, Japan). Axial length (AL) measurements were obtained using the OA-2000 (TOMEY, Aichi, Japan). All SD-OCT measurements were carried out after pupil dilation with 1% tropicamide and OCT imaging was performed using the laser scan protocol. Data with apparent eye movement and involuntary blinking or saccade during the measurement were carefully excluded. Following the manufacturer’s recommendation, imaging data with quality factor <7 were also excluded. The fundus photograph and the photoreceptor inner segment/outer segment line and the retinal pigment epithelium (RPE) on the OCT image were carefully reviewed by a specialist in macular disease (T.I.) so that subjects with other retinal disease could be excluded from the study. Figure 1 shows the RNFL, GCL + IPL, inner nuclear layer (INL) + outer plexiform layer (OPL), outer nuclear layer (ONL) + IS layer (ISL) and OSL + RPE in a sample case. Similar to our previous report analyzing OCT data^11^, the fovea was automatically identified as the pixel with thinnest retinal thickness close to the fixation point, and a square imaging area (9.0 × 9.0 mm) was centered on the fovea, excluding the area of the optic disc and parapapillary atrophy. Using software supplied by the manufacturer, thicknesses of (i) RNFL, (ii) GCL + IPL, (iii) INL + OPL, (iv) ONL + ISL and (v) OSL + RPE (see Fig. 1) were exported. These were exported as 512 × 128 pixel images, and the mean thickness values of the whole analysis area (9.0 × 9.0 mm, corrected with AL) were calculated.

### Statistical analysis

The structure-function relationship between mTH and the variables of age, AL, and the RNFL thickness was analyzed using the linear mixed model, whereby each subject was treated as a random effect (model_all__RNFL). Similarly, the structure-function relationship between mTH and the variables of age, AL, and the GCL + IPL thickness was analyzed (model_all__GCLIPL). Then, the structure-function relationship between mTH and the variables of age, AL, and the total thickness of RNFL and GCL + IPL was analyzed (model_all__RNFL_GCLIPL). Subsequently, the structure-function relationship was evaluated by supplementing the total thickness of RNFL and GCL + IPL with: (i) INL + OPL (model_all__RNFL_GCLIPL_INLOPL); (ii) ONL + ISL (model_all__RNFL_GCLIPL_ONLISL); (iii) OSL + RPE (model_all__RNFL_GCLIPL_OSLRPE). Models were compared using the second-order bias-corrected Akaike Information Criterion (AICc) index. The AICc is an established statistical measure to evaluate model fit, and the AICc is a corrected type of AICc, which provides an accurate estimation even when the sample size is small^18^. The marginal R-squared (mR^2^) value was calculated following a method proposed by Nakagawa and Holger^19^. Any magnitude of reduction in AICc suggests an improvement of the model fit, and the probability that a particular model minimizes ‘information loss’ can be calculated as follows^20^; when there are *n* candidate models and AICc values of those models are AIC_1_, AIC_2_, AIC_3_, …, AIC_n_. If AIC_min_ is the minimum of these value then exp((AIC_min_ − AIC_*i*_)/2) describes the relative probability (RP) that *i*th model minimizes the information loss (i.e. it is the ‘optimal model’).

The structure-function relationship was also investigated in subsets of the whole study population. The sample was divided into four groups using refractive error and presence of glaucoma. Refractive error between −6.0 and +3.0 diopter was defined as the non-highly myopic group and refractive error less than −6.0 diopter was defined as the highly myopic group. The four groups of eyes were thus labelled as: non-highly myopic and glaucomatous (‘G’ group); highly myopic and glaucomatous (‘Gm’ group); non-highly myopic and normative (‘N’ group); highly myopic and normative (‘Nm’ group).

All statistical analyses were carried out using the statistical programming language R (ver. 3.1.3, The R Foundation for Statistical Computing, Vienna, Austria).

## Results

Subject characteristics are given in Table 1. Among 615 eyes of 391 subjects, 356 eyes of 231 patients were non-highly myopic and glaucomatous, 159 eyes of 107 patients were highly myopic and glaucomatous, 74 eyes of 39 subjects were non-highly myopic and normative, 26 eyes of 14 subjects were highly myopic and normative.

**Table 1:** Demographic data.

Variables	Value
Age, (mean ± sd)[range], years old	54.68 ± 15.21 [23 to 79]
glaucoma/normal	338/53
male/female	170/221
right/left	310/305
AL, (mean ± sd)[range], mm	25.46 ± 1.66 [21.73 to 31.33]
Refraction, (mean ± sd)[range], Diopter	−4.06 ± 3.52 [−14.0 to 4.0]
mTH, (mean ± sd)[range], dB	26.10 ± 8.19 [0.54 to 35.87]

sd: standard deviation, AL: axial length, mTH: mean of total visual threshold value.

As shown in Table 2, across all eyes, the thicknesses of RNFL, GCLIPL, RNFL + GCLIPL, RNFL + GCLIPL + INLOPL, RNFL + GCLIPL + ONLISL and RNFL + GCLIPL + OSLRPE were significantly related to mTH (p < 0.001, linear mixed model). The AICc values of model_all__RNFL and model_all__GCLIPL were 3962.7 and 4018.5, respectively, whereas, that of model_all__RNFL_GCLIPL was 3851.0. The AICc values of model_all__RNFL_GCLIPL_INLOPL and model_all__RNFL_GCLIPL_ONLISL were 3901.5 and 3947.7, respectively. The optimal model according to the AICc test statistic was model_all__RNFL_GCLIPL_OSLRPE (AICc = 3832.9). The mR^2^ value associated with model_all__RNFL_GCLIPL was 48.7%, whereas that with model_all_ RNFL_GCLIPL_OSLRPE was 50.4%. The relative probability that model_all__RNFL_GCLIPL_OSLRPE was optimal compared to model_all__RNFL_GCLIPL was 0.9999.

**Table 2:** Structure-function relationship between the mean of the whole field’s threshold values and inner retinal layer thickness with adjustment for age and axial length

Variables	mR^2^	p value	AICc	RR
model_all__RNFL	0.43	<0.001	3962.7	6.5 × 10^−29^
model_all__GCLIPL	0.35	<0.001	4018.5	5.0 × 10^−41^
model_all__RNFL_GCLIPL	0.53	<0.001	3851.0	1.2 × 10^−4^
model_all__RNFL_GCLIPL_INLOPL	0.48	<0.001	3901.5	1.3 × 10^−15^
model_all__ RNFL_GCLIPL_ONLISL	0.43	<0.001	3947.7	1.2 × 10^−25^
model_all__ RNFL_GCLIPL_OSLRPE	0.54	<0.001	3832.9	—

mR^2^: Marginal R-squared value calculated with a method by Nakagawa and Holger^19^, AICc: The second-order bias-corrected Akaike Information Criterion, RNFL: retinal nerve fiber layer, GCL: ganglion cell layer, IPL: inner plexiform layer, INL: inner nuclear layer, OPL: outer plexiform layer, ONL: outer nuclear layer, IS: inner segment of photoreceptor layer, OSL: outer segment of photoreceptor layer, RPE: retinal pigment epithelium,

RNFL_GCLIPL: total thickness of retinal nerve fiber layer, ganglion cell layer + inner plexiform layer,

RNFL_GCLIPL_ INLOPL: total thickness of retinal nerve fiber layer, ganglion cell layer + inner plexiform layer and inner nuclear layer + outer plexiform layer,

RNFL_GCLIPL_ ONLISL: total thickness of retinal nerve fiber layer, ganglion cell layer + inner plexiform layer and outer nuclear layer + inner segment of photoreceptor,

RNFL_GCLIPL_OSLRPE: total thickness of retinal nerve fiber layer, ganglion cell layer + inner plexiform layer and outer segment of photoreceptor + retinal pigment epithelium.

In group G, as shown in Table 3, RNFL, GCLIPL, RNFL + GCLIPL, RNFL + GCLIPL + INLOPL, RNFL + GCLIPL + ONLISL and RNFL + GCLIPL + OSLRPE were significantly related to mTH (p < 0.001, linear mixed model). The AICc values of model_G__RNFL and model_G__GCLIPL was 2310.8 and 2305.2, respectively, whereas, the AICc value of model_G__RNFL_GCLIPL was 2209.4. The AICc values of the remaining models were: model_G__RNFL_GCLIPL_INLOPL (AICc = 2228.7), model_G__RNFL_GCLIPL_ONLISL (AICc = 2274.2), model_G__RNFL_GCLIPL_OSLRPE (AICc = 2199.5). The mR^2^ values associated with model_G__RNFL_GCLIPL was 48.8%, whereas that with model_G_ RNFL_GCLIPL_OSLRPE was 50.8%. The relative probability that model_G__RNFL_GCLIPL_OSLRPE was optimal compared to model_G__RNFL_GCLIPL was 0.993.

**Table 3:** Structure-function relationship between the mean of the whole field’s threshold values and retinal layer thickness with adjustment for age and axial length, in glaucomatous and normative eyes with and without high myopia.

Model	mR^2^	p value	AICc	Relartive probability	model	mR^2^	p value	AICc	Relartive probability
**G**					**Gm**				
model_G__RNFL	0.31	<0.001	2310.8	6.8 × 10^−25^	model_Gm__RNFL	0.34	<0.001	1063.9	2.0 × 10^−4^
model_G__GCLIPL	0.30	<0.001	2305.2	1.1 × 10^−23^	model_Gm__GCLIPL	0.13	<0.001	1102.9	6.9 × 10^−13^
model_G__RNFL_GCLIPL	0.49	<0.001	2209.4	7.1 × 10^−3^	model_Gm__RNFL_GCLIPL	0.37	<0.001	1053.5	0.037
model_G__RNFL_GCLIPL_INLOPL	0.45	<0.001	2228.7	4.6 × 10^−7^	model_Gm__RNFL_GCLIPL_INLOPL	0.28	<0.001	1075.0	7.9 × 10^−7^
model_G__RNFL_GCLIPL_ONLISL	0.37	<0.001	2274.2	6.0 × 10^−17^	model_Gm__RNFL_GCLIPL_ONLISL	0.26	<0.001	1076.8	3.2 × 10^−7^
model_G__RNFL_GCLIPL_OSLRPE	0.51	<0.001	2199.5	—	model_Gm__RNFL_GCLIPL_OSLRPE	0.40	<0.001	1046.9	—
**N**					**Nm**				
model_N__RNFL	0.074	0.054	170.7		model_Nm__RNFL	0.17	0.35	70.8	0.14
model_N__GCLIPL	0.030	0.43	174.2		model_Nm__GCLIPL	0.16	0.76	71.5	0.10
model_N__RNFL_GCLIPL	0.069	0.11	172.0		model_Nm__RNFL_GCLIPL	0.22	0.086	70.4	0.17
model_N__RNFL_GCLIPL_INLOPL	0.061	0.14	172.4		model_Nm__RNFL_GCLIPL_INLOPL	0.19	0.22	70.4	0.17
model_N__RNFL_GCLIPL_ONLISL	0.037	0.34	173.9		model_Nm__RNFL_GCLIPL_ONLISL	0.16	0.43	71.5	0.095
model_G__RNFL_GCLIPL_OSLRPE	0.077	0.077	171.5	—	model_Gm__RNFL_GCLIPL_OSLRPE	0.33	0.001	66.8	—

G: glaucoma eyes (between −6.0 and +3.0 diopter), Gm: glaucoma eyes (less than −6.0 diopter), N: normal eyes (between −6.0 diopter and +3.0 diopter), Nm: normal eyes (less than −6.0 diopter), mR^2^: Marginal R-squared value calculated with a method by Nakagawa and Holger^19^, AICc: The second-order bias-corrected Akaike Information Criterion, RNFL_GCLIPL: total thickness of retinal nerve fiber layer, ganglion cell layer + inner plexiform layer, RNFL_GCLIPL_ INLOPL: total thickness of retinal nerve fiber layer, ganglion cell layer + inner plexiform layer and inner nuclear layer + outer plexiform layer, RNFL_GCLIPL_ ONLISL: total thickness of retinal nerve fiber layer, ganglion cell layer + inner plexiform layer and outer nuclear layer + inner segment of photoreceptor, RNFL_GCLIPL_OSLRPE: total thickness of retinal nerve fiber layer, ganglion cell layer + inner plexiform layer and outer segment of photoreceptor + retinal pigment epithelium, relative probability was not calculated in group N, since the model_N__RNFL had a smaller AICc than model_N__RNFL_GCLIPL_INLOPL.

As shown in Table 3, in group Gm, RNFL, GCLIPL, RNFL + GCLIPL, RNFL + GCLIPL + INLOPL, RNFL + GCLIPL + ONLISL and RNFL + GCLIPL + OSLRPE were significantly related to mTH (p < 0.001, linear mixed model). The AICc values of model_Gm__RNFL and model_Gm__GCLIPL was 1063.9 and 1102.9, respectively, whereas, the AICc value of model_Gm__RNFL_GCLIPL was 1053.5. The model_Gm__RNFL_GCLIPL_OSLRPE had a lower AICc value (1046.9) compared to model_Gm__RNFL_GCLIPL. Other models of model_Gm__RNFL_GCLIPL_INLOPL and model_Gm__RNFL_GCLIPL_ONLISL had larger AICc values (1075.0 and 1076.8) than model_Gm__RNFL_GCLIPL. The mR^2^ values associated with model_Gm__RNFL_GCLIPL was 37.4%, whereas that with model_Gm_ RNFL_GCLIPL_OSLRPE was 40.2%. The relative probability that model_Gm__RNFL_GCLIPL_OSLRPE was optimal compared to model_Gm__RNFL_GCLIPL was 0.96.

As shown in Table 3, in group N, RNFL and GCLIPL were not significantly related to mTH (p = 0.054 and 0.43, linear mixed model), and likewise, total thickness of RNFL + GCLIPL, RNFL + GCLIPL + INLOPL, RNFL + GCLIPL + ONLISL and RNFL + GCLIPL + OSLRPE were not significantly related to mTH (p = 0.11, 0.14, 0.34 and 0.077, respectively, linear mixed model). The AICc values of model_N__RNFL and model_N__GCLIPL were 170.7 and 174.2, respectively, whereas, the AICc value of model_N__RNFL_GCLIPL was 172.0. The model_N__RNFL_GCLIPL_OSLRPE had a lower AICc value (171.5) compared to model_N__RNFL_GCLIPL. Other models of model_N__RNFL_GCLIPL_INLOPL and model_N__RNFL_GCLIPL_ONLISL had larger AICc values (172.4 and 173.9). The mR^2^ values associated with model_N__RNFL_GCLIPL was 6.9%, whereas that with model_N_ RNFL_GCLIPL_OSLRPE was 7.7%.

As shown in Table 3, in group Nm, RNFL and GCLIPL were not significantly related to mTH (p = 0.35 and 0.76, respectively, linear mixed model), and likewise, total thickness of RNFL + GCLIPL, RNFL + GCLIPL + INLOPL and RNFL + GCLIPL + ONLISL were not significantly related to mTH (p = 0.086, 0.22 and 0.43, respectively, linear mixed model). However, RNFL + GCLIPL + OSLRPE was related to mTH (p = 0.001, linear mixed model). The AICc values of model_Nm__RNFL and model_Nm__GCLIPL were 70.8 and 71.5, respectively, whereas, the AICc value of model_Nm__RNFL_GCLIPL was 70.4. model_Nm__RNFL_GCLIPL_OSLRPE had a lower AICc value (66.8) compared to model_Nm__RNFL_GCLIPL. Other remaining models of model_Nm__RNFL_GCLIPL_INLOPL and model_Nm__RNFL_GCLIPL_ONLISL had larger AICc values (70.4 and 71.5, respectively). The mR^2^ value associated with model_Nm__RNFL_GCLIPL was 21.7%, whereas that with model_Nm_ RNFL_GCLIPL_OSLRPE was 33.4%. The relative probability that model_Nm__RNFL_GCLIPL_OSLRPE was optimal compared to model_Nm__RNFL_GCLIPL was 0.8 (see Table 3).

The relationship between OSL + RPE thickness and: (i) RNFL thickness and (ii) GCL + IPL thickness, with adjustment for age and AL, in the groups of G and Gm is shown in Table 4. The OSL + RPE thickness was significantly correlated to RNFL thickness in groups G (coefficient = −0.07 and p = 0.0016, linear mixed model) and Gm (coefficient = −0.07 and p = 0.032). However, this significant relationship was not observed in groups N and Nm (p = 0.65 and 0.35, respectively). In contrast, OSL + RPE thickness was not significantly related to GCL + IPL thickness in all groups (group G: p = 0.15, group Gm: p = 0.53, group N: p = 0.10 and group Nm: p = 0.58).

**Table 4:** The relationship between outer segment layer + retinal pigment epithelium thickness and: (i) retinal nerve fiber layer thickness and (ii) ganglion cell layer + inner plexiform layer thickness.

	RNFL		GCL + IPL	
	mR^2^	p value	mR^2^	p value
Group G	0.20	0.0016	0.18	0.15
Group Gm	0.071	0.032	0.048	0.53

Group G: between −6.0 diopter and +3.0 diopter in eyes with glaucoma.

Group Gm: less than −6.0 diopter in eyes with glaucoma.

RNFL: retinal nerve fiber layer, GCL: ganglion cell layer, IPL: inner plexiform layer.

## Discussion

In the current study, OCT and HFA 10-2 VF measurements were carried out in glaucomatous and normative eyes, with and without high myopia. The total thickness of RNFL, GCL + IPL and OSL + RPE was more strongly correlated with VF sensitivity than the total thickness of RNFL and GCL + IPL in all subgroups, in particular in glaucomatous groups. Including the thickness of other layers, namely INL + OPL, or, ONL + ISL, resulted in a weaker relationship compared to the total thickness of RNFL and GCL + IPL in all subgroups. There was a significant correlation between OSL + RPE thickness and RNFL thickness in glaucomatous eyes (regardless of high myopia) but not in normative eyes.

Numerous studies have investigated the structure-function relationship using the thicknesses of RNFL and GCL + IPL and HFA VF testing^21–23^. In these studies the VF was measured using either the 24-2 or 30-2 test pattern, whereas the macular scanning region of OCT corresponds to a much narrower area of the VF^9^, making the HFA 10-2 test pattern more relevant for investigating the structure-function relationship. There are a limited number of studies that analyzed the relationship between the 10-2 HFA VF and RNFL or GCL + IPL thickness. Ohkubo *et al*. suggested a significant correlation between mean 10-2 VF sensitivity and RNFL (r = 0.759, p < 0.0001, Spearman correlation coefficient), and also GCL + IPL (r = 0.520, p < 0.001) in glaucoma patients^24^. Takahashi *et al*. suggested that mean VF sensitivity measured with HFA 10-2 is significantly correlated to RNFL thickness (r = 0.76, p < 0.001) and GCL + IPL thickness (r = 0.55, p < 0.001), and a similar magnitude of strength was observed in the structure-function relationship between VF sensitivity and RNFL and GCL + IPL thickness (r = 0.72, p < 0.001)^25^. In the current study, in non-highly myopic glaucomatous eyes (group G), RNFL and GCL + IPL were significantly correlated to VF sensitivity, however, a considerable decrease in AICc was observed by using the total thickness of RNFL and GCL + IPL (AICc values were 2310.8 and 2305.2 vs. 2209.4, respectively). This finding was also observed in all subgroups of eyes (see Table 3).

Numerous reports have observed a decreased inner retinal layer thickness of RNFL and GCL + IPL in glaucomatous eyes^8,21,26–31^; however, the involvement of the outer segment of the retina in the disease process remains controversial. In histological studies, Panda and Jonas reported that PhR count was significantly lower in secondary angle closure glaucoma than in control eyes^32^. Nork *et al*. investigated the postmortem eyes with a diagnosis of chronic glaucoma and suggested loss of PhR in POAG eyes^33^. However, in Kendell *et al*.^34^, the number and density of PhR in postmortem eyes were not significantly different between glaucoma and age-matched control eyes. Thus, the involvement of PhR in the development and progression of glaucoma is undetermined. In the current study, a significant relationship was observed between OSL + RPE thickness and RNFL thickness in the non-highly myopic glaucoma group, which suggests the involvement of PhR in the disease.

With the development of SD-OCT, it is now possible to measure the thicknesses of the inner and outer retina accurately and noninvasively, however, the association between the thickness of the outer retinal layer thickness measured with SD-OCT and VF sensitivity in glaucoma has not been investigated in detail. In our previous report, the effect of PhR layer thickness measured with SD-OCT on VF sensitivity in glaucoma was investigated, and it was suggested that OSL + RPE thickness was significantly correlated with VF sensitivity (coefficients = 0.63, p < 0.001), independent from the thickness of RNFL and GCL + IPL^11^. Supporting this, one previous study suggested structural changes in cone PhR, such as shortening and/or swelling OSL, where VF sensitivity was compromised, in glaucoma^35^. However, in this study, the outer segment of the retina was scanned using OCT in only ten eyes. In the current study, the structure-function relationship was investigated in a much larger population (615 eyes in total). We observed that the structure-function relationship was stronger between the total thickness of RNFL, GCL + IPL and OSL + RPE, and 10-2 HFA VF sensitivity, compared to that between the total thickness of RNFL and GCL + IPL, and 10-2 HFA VF sensitivity. This finding was also observed in the group of Nm, suggesting the importance of OSL + RPE thickness on visual function is not only due to the possible involvement of PhR in the disease process of glaucoma, and a thick PhR is advantageous in normative highly myopic eyes, although this tendency was much weaker in normative eyes than in glaucomatous eyes. Furthermore, in glaucomatous eyes, the OSL + RPE thickness may also be associated with the advancement of glaucoma, as suggested by the significant relationship between OSL + RPE thickness and RNFL thickness.

There are many previous studies which investigated the structure-function relationship in glaucoma^1–4,11^, however, these studies were usually performed excluding highly myopic eyes. The prevalence of glaucoma is elevated in highly myopic eyes^36–40^. In myopia, the optic disc is deformed because of the elongation of the eye^41^. Analyzing the structure-function relationship in glaucoma in conjunction with the degree of myopia is very important, particularly in investigating the disease mechanism of glaucoma, because highly myopic eyes and glaucomatous eyes share similar features. For instance, peripapillary atrophy (PPA) is associated with the development and the progression of glaucoma^42–49^, but PPA is also commonly seen in myopic eyes^50–52^, so the nature of PPA may be different in glaucoma and in myopia^53,54^. Miki *et al*. reported that prevalence of a lamina cribrosa defect can be observed both in glaucomatous and highly myopic eyes^55^. Nonetheless, previous studies on the structure-function relationship in glaucoma have usually been conducted excluding eyes without high myopia. In highly myopic eyes, RNFL^56–63^, GCL + IPL^64,65^ and OSL + RPE thicknesses^66^ decrease with elongation of an eye. It should be noted, in turn, that myopia may cause visual dysfunction such as a decline of visual acuity^67,68^, high contrast visual acuity^69^, contrast sensitivity^70^, and also spatial summation^71^. Thus, the effects of myopic change on the glaucomatous structure-function relationship is complex. Our results suggest that total thickness of RNFL, GCL + IPL and OSL + RPE is more strongly correlated to VF sensitivity than the total thickness of RNFL and GCL + IPL regardless of glaucoma status and degree of myopia. This suggests that PhR thickness is associated with both glaucomatous and myopic changes.

As suggested in a review by Hood^72^, a single scan of OCT has the potential to replace the VF measurement, however, the VF test is not redundant yet. Significant efforts are required to improve the structure-function relationship but modern statistical techniques, such as a deep learning, have shown promise to predict VF sensitivity accurately from OCT measurements^73^. In these precision algorithms, even a small improvement of the input data could result in a huge improvement in the outcome.

One of the limitations of the current study is a limited number of normal subjects, compared to glaucomatous eyes. A further study should be carried out increasing the number of such eyes. Also, the current study used the results of the segmentation software equipped in RS3000. The results should be confirmed using OCTs from other manufacturers.

In conclusion, the structure-function relationship was highly more optimal when OSL + RPE thickness is considered in addition to RNFL, GCL and IPLin highly myopic glaucomatous eyes, non-highly myopic glaucomatous eyes, and highly-myopic normative eyes. The OSL + REP thickness was correlated to RNFL thickness in glaucoma, with and without high myopia.
